# Species-specific modifications of mandible shape reveal independent mechanisms for growth and initiation of the coronoid

**DOI:** 10.1186/s13227-015-0030-6

**Published:** 2015-11-14

**Authors:** Neal Anthwal, Heiko Peters, Abigail S. Tucker

**Affiliations:** Department of Craniofacial Development and Stem Cell Biology, Dental Institute, King’s College London, London, SE1 9RT UK; Institute of Genetic Medicine, International Centre for Life, Newcastle University, Newcastle upon Tyne, NE1 3BZ UK

**Keywords:** Mandible, Dentary, Mammal, Mammalian evolution, Coronoid process, Pax9, Sox9, Guinea pig, Opossum mouse

## Abstract

**Background:**

The variation in mandibular morphology of mammals reflects specialisations for different diets. Omnivorous and carnivorous mammals posses large mandibular coronoid processes, while herbivorous mammals have proportionally smaller or absent coronoids. This is correlated with the relative size of the temporalis muscle that forms an attachment to the coronoid process. The role of this muscle attachment in the development of the variation of the coronoid is unclear.

**Results:**

By comparative developmental biology and mouse knockout studies, we demonstrate here that the initiation and growth of the coronoid are two independent processes, with initiation being intrinsic to the ossifying bone and growth dependent upon the extrinsic effect of muscle attachment. A necessary component of the intrinsic patterning is identified as the paired domain transcription factor Pax9. We also demonstrate that Sox9 plays a role independent of chondrogenesis in the growth of the coronoid process in response to muscle interaction.

**Conclusions:**

The mandibular coronoid process is initiated by intrinsic factors, but later growth is dependent on extrinsic signals from the muscle. These extrinsic influences are hypothesised to be the basis of the variation in coronoid length seen across the mammalian lineage.

## Background

**Fig. 1:**
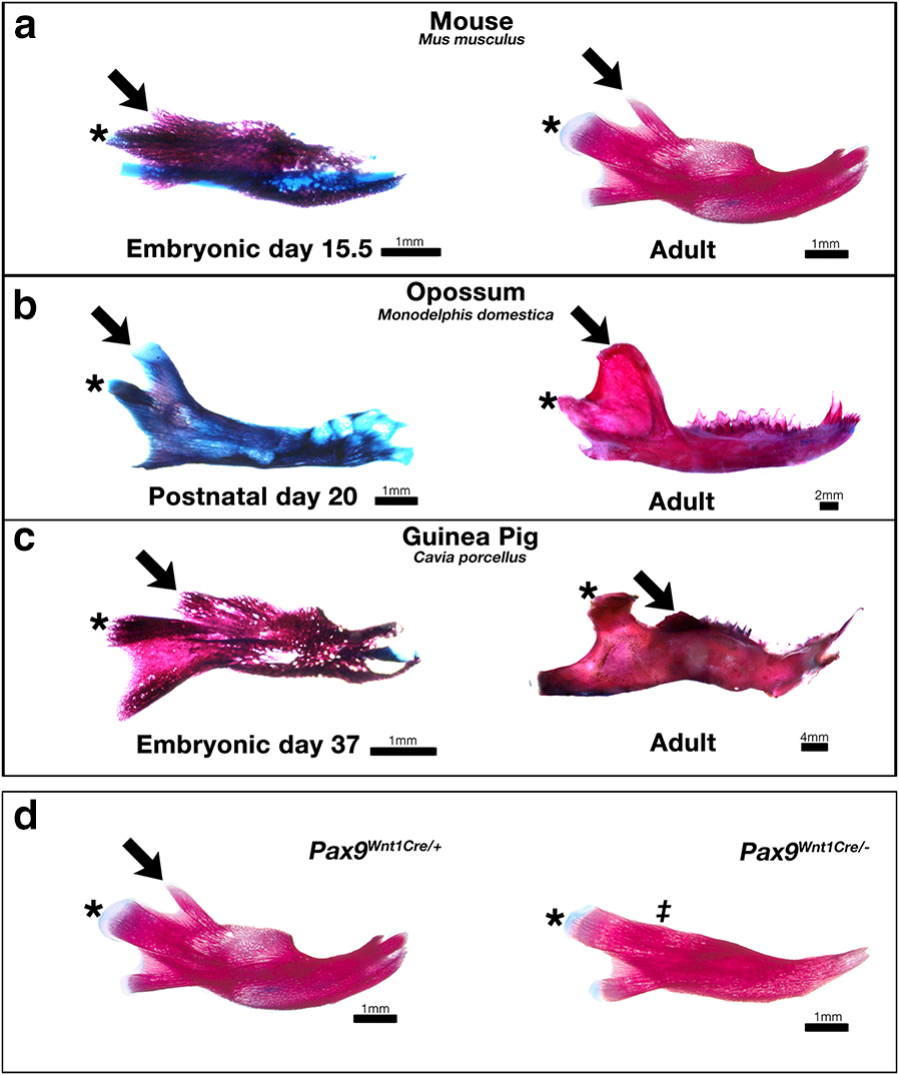
Alizarin *red*/Alcian *blue* staining of guinea pig, mouse and opossums mandibles. **a**–**c** The coronoid process is a distinct part of the dentary in mammals such as the mouse (*Mus musculus*) and opossum (*Mondelphis domestica*) during development and in the adult (**a**, **b**), whereas the guinea pig (*Cavia* *porcellus*) displays a coronoid process comparable with the mouse in embryonic stages (E37 presented in **c**), but only has a rudimentary process in adults. **d** Conditional deletion of *Pax9* in neural crest lineage cells results in a loss of the coronoid process [8]; *Arrowheads*: location of coronoid process; *asterisk* location of condylar process, *double dagger* location of absent coronoid process

Mammals have adapted to a range of different ecological niches, including a wide variety of diets from the purely carnivorous to exclusively herbivorous. To adapt to these various lifestyles, the skeletal elements associated with feeding have changed in size and shape during evolution. Many of these elements, such at the teeth and skeleton of the jaws are among the defining characteristic of mammals [1]. In contrast to other gnathostomes, the mammalian mandible comprises a single bone (the dentary) that has undergone an increase in complexity and modularity during evolution [2]. The coronoid process, which is the site of attachment of the temporalis muscle, is one of the morphological units of the dentary that offers insight into the adaptations that have occurred during mammalian evolution. Following the late cretaceous angiosperm radiation (100.5 million years ago), an increased number of plant and fruit based dietary niches were made available to early mammals. Adaptation to a more herbivorous diet resulted in changes in the size and shape of the mandible bone such that extant carnivorous, omnivorous, and insectivorous mammals, including mice and Didelphidae opossums (Fig. 1a, b) have a large coronoid process, reflecting the basal mammalian form, while herbivores have a reduced process [3]. Extreme versions of this form can be observed in grass- and leaf-eating mammals such as guinea pigs, where bite force is lower compared with other rodents [4] and the coronoid process is relatively small in size such that it appears absent in many adults (Fig. 1c) [5].

How the variability between species is developmentally patterned is currently unknown. In the mouse, the basic shape of the dentary, including the proximal coronoid, condylar and angular processes, is established before the onset of ossification at embryonic day 13.5 [6], suggesting an intrinsic mechanisms of dentary patterning. Insights into the genetic control of dentary patterning have been gained through the use of mutant mouse lines (see [5] for further review). Conditional deletions of *Sox9* and *Pax9*, specifically in neural crest cells (NCC), which make up the bone of the mandible, as well as a homozygous Bmp2 deletion with a heterozygous Bmp4 deletion in the same population, each result in loss of the coronoid process [7–9]. External effectors such as muscle attachments, however, are also known to have an influence upon the shape of the dentary. Classical studies involving post-natal surgical manipulation of a range of experimental animals have demonstrated that alterations in muscle attachment result in a change in coronoid size and shape [10–17]. In the modern era mouse knockout studies have supported this finding, whilst allowing for developmental effects to be explored. Mice lacking the myogenic factors *Myf5* and *MyoD* have severely disrupted dentary bones at E18.5, including a reduction or loss of the coronoid and angular processes [18], and a similar phenotype is observed in *Tbx1* mutant mice that lack the mesoderm derived muscles of the head [19].

While these studies have indicated that intrinsic patterning and extrinsic interactions with muscle affect the coronoid process, no study has specifically addressed which factors and developmental mechanisms control coronoid process development. Questions remain as to how and when *Pax9* controls coronoid development, and whether extrinsic factors are required for the initial patterning of the dentary or if the basic shape is independent of external influence.

The current study demonstrates that intrinsic expression of *Pax9* is required for the induction of the coronoid process before the ossification of the dentary at E14.5. We demonstrate that mammals with large variations in relative coronoid size as adults all initially develop a coronoid process with expression of *Pax9* in the mesenchyme that gives rise to the bone. We show that subsequent variation is dependent on extrinsic differences in muscle, suggesting that the developing temporalis muscle is required to maintain and grow the coronoid process. Finally, we suggest a role for *Sox9* in the growth of the coronoid in response to muscle, independent of its canonical role in cartilage development.

## Methods

### Animals and tissue processing

In all mouse lines, timed matings were produced by observation of a vaginal plug following overnight mating, midday on the day the plug found being considered embryonic day 0.5 (E0.5). *Wnt1*-*Cre*^*Cre/*+^*;Pax9*^*flox/flox*^ (named here *Pax9*^*Wnt1Cre/*−^) mice and *Wnt1*-*Cre*^+*/*+^*;Pax9*^*flox/flox*^ (*Pax9*^*Wnt1Cre/*+^) wildtype littermates were generated as previously described [7]. *Pax9*^*LacZ/*+^ heterozygous reporter mice (named here *Pax9*^*LacZ*^) were generated as previously described [11]. The generation of a conditional *Tbx1* deletion in mesodermal lineage cells was carried out by crossing Tbx1^flox/flox^ female mice with Mesp1-Cre^Cre/+^ males [12, 13]. The resulting male Tbx1^flox/+^;Mesp1-Cre^Cre/+^ progeny were crossed with Tbx1^flox/flox^ females to generate conditional mutant embryos and wildtype (Tbx1^flox/flox^;Mesp1-Cre^+/+^) littermates; here named *Tbx1*^*Mesp1Cre/*−^ and *Tbx1*^*Mesp1Cre/*+^, respectively.

Mated guinea pigs were acquired from Harlan Laboratories, UK. Timed matings were carried out immediately following the littering down of a previous mating.

Grey short tailed opossum (*Monodelphis domestica*) pups were supplied by the laboratory of Dr James Turner at the Francis Crick Institute, Mill Hill Laboratory. Matings were confirmed by video observation of mating pairs, and the date of birth calculated from date of mating.

Experimental animals were cared for and killed according to UK Home Office licence and regulations in line with the regulations set out under the United Kingdom Animals (Scientific Procedures) Act 1986 and the European Union Directive 2010/63/EU.

All tissues for histological sectioning were fixed overnight at 4 °C in 4 % paraformaldehyde (PFA), before being dehydrated through a series of graded methanol followed by isopropanol, cleared with 1234-tetrahydronaphthalene, before wax infiltration with paraffin wax at 60 °C. All solutions used were made nuclease free by overnight treatment with 0.1 % diethylpyrocarbonate (DEPC) followed by neutralisation by autoclaving. Wax embedded samples were microtome sectioned at 8 µm thickness, then mounted in parallel series on charged slides. The first slide from each series was stained with haematoxylin/eosin, or pico-sirius red/alcian blue trichrome using standard techniques, with the remaining slides from each series used for immunohistochemistry and in situ hybridisation.

Samples for alizarin red, alcian blue skeletal preparations were skinned and eviscerated when appropriate, before fixation in 95 % ethanol. Staining was then carried out as previously described [6].

### Immunohistochemistry, X-Gal staining, and in situ hybridisation

For immunofluorescence staining for Sox9 in mouse and guinea pig, and with muscle specific 12/101 antibody in mouse, slides were rehydrated through a graded series of ethanols to PBS. Heat induced antigen retrieval was carried out by microwaving the samples for 10 min in 0.1M Sodium citrate buffer. Slides were then blocked in 1 % Bovine serum albumin, 0.1 % cold water fish skin gelatine, 0.1 % triton-X for 1 h. Sections were then treated over night at 4 °C with rabbit polyclonal antibody against human Sox9 (Chemicon AB5535) diluted 1/200 in blocking buffer, or 12/101 mouse monoclonal antibody (DSHB) diluted 1/100 in the blocking buffer. Following repeated PBS washes, secondary antibody (Alexa568 conjugated Donkey anti-Rabbit—Invitrogen) was added to the Sox9 slides at 1/300 in the blocking buffer for 1 h at room temperature. The secondary antibody was then washed off with PBS, and the slides mounted with Fluroshield mounting medium containing DAPI (Abcam). Sections were visualised by Leica SP5 confocal microscopy. For 12/101 slides, biotinylated goat anti-mouse antibody (Dako) was added to the slides 1/400 in blocking buffer. Slides were then washed in PBS before being treated with ABC-HRP streptavidin kit (Vector Labs), and then revealed with DAB (Vector Labs).

X-Gal staining for LacZ expressing cells on sections of *Pax9*^*LacZ*^ mouse mandibles was carried out as previously described [20].

For the detection of *Pax9* mRNA expression in guinea pig, a 550 bp fragment of guinea pig *Pax9* [GenBank: XM_013154571] was cloned by PCR from RNA extracted from E28 thoracic tissue using the following primers: Fwd–CGTGTGCGACAAGTACAACG, Rev–GCAGCGCTGTAGGTCATGTA. This fragment was then ligated into pJet1.2 vector plasmid using the CloneJeT PCR cloning kit (Thermo Scientific) and transformed into DH5α competent cells. Clonal colonies containing the guinea pig *Pax9* fragment in the antisense direction to the T7 transcription site were selected by colony PCR using the forward gp*Pax9* primer and the pJet1.2 forward sequencing primer: CGACTCACTATAGGGAGAGCGGC. Following midiprep culture and plasmid extraction (Qiagen) antisense probes were transcribed from the p*Jet1.2*-*gpPax9* plasmid. Antisense probes were also transcribed from plasmids containing mouse *Scx* [21]*, Col2a,* or *Pax 9,* and opossum *(Monodelphis)Pax9* (a gift from the Sears lab, University of Illinois, Urbana-Champaign). In situ hybridization was then carried out on paraffin wax sections by previously published techniques [22]. All histology and expression studies were duplicated at least once.

### Muscle measurements

Estimates of temporalis muscle volume were performed on histologically stained E15.5 *Tbx1*^*Mesp1Cre/*+^ and *Tbx1*^*Mesp1Cre/*−^ sagittal sections (*n* = 3). The area of the muscle was measured every 4th section (i.e. every section of a single parallel series for each individual) using ImageJ, and the volume estimated by multiplying each area by the tissue depth between each series (32 µm). The total muscle volume was then estimated by the summing of these values. *T*-test comparison between *Tbx1*^*Mesp1Cre/*−^ and *Tbx1*^*Mesp1Cre/*+^ was made using Prism 6 software (Graphpad).

### Explant culture

Dentary bone and mesenchyme from mice aged E14.5 and E15.5 were cultured as previously described [6], with some modifications. E14.5 explants were cultured in BGJb medium with the addition of 10 % foetal bovine serum (FBS, *n* = 16), while control explants were cultured in serum free conditions (*n* = 10). E15.5 explants were cultured in BGJb medium with 10 % FBS (*n* = 42). Following culture, explants were fixed in 4 % PFA, before dehydration for histological analysis. Throughout these experiments FBS was used which had not been heat inactivated.

## Results

### *Pax9* is required for development of the dentary coronoid process

**Fig. 2:**
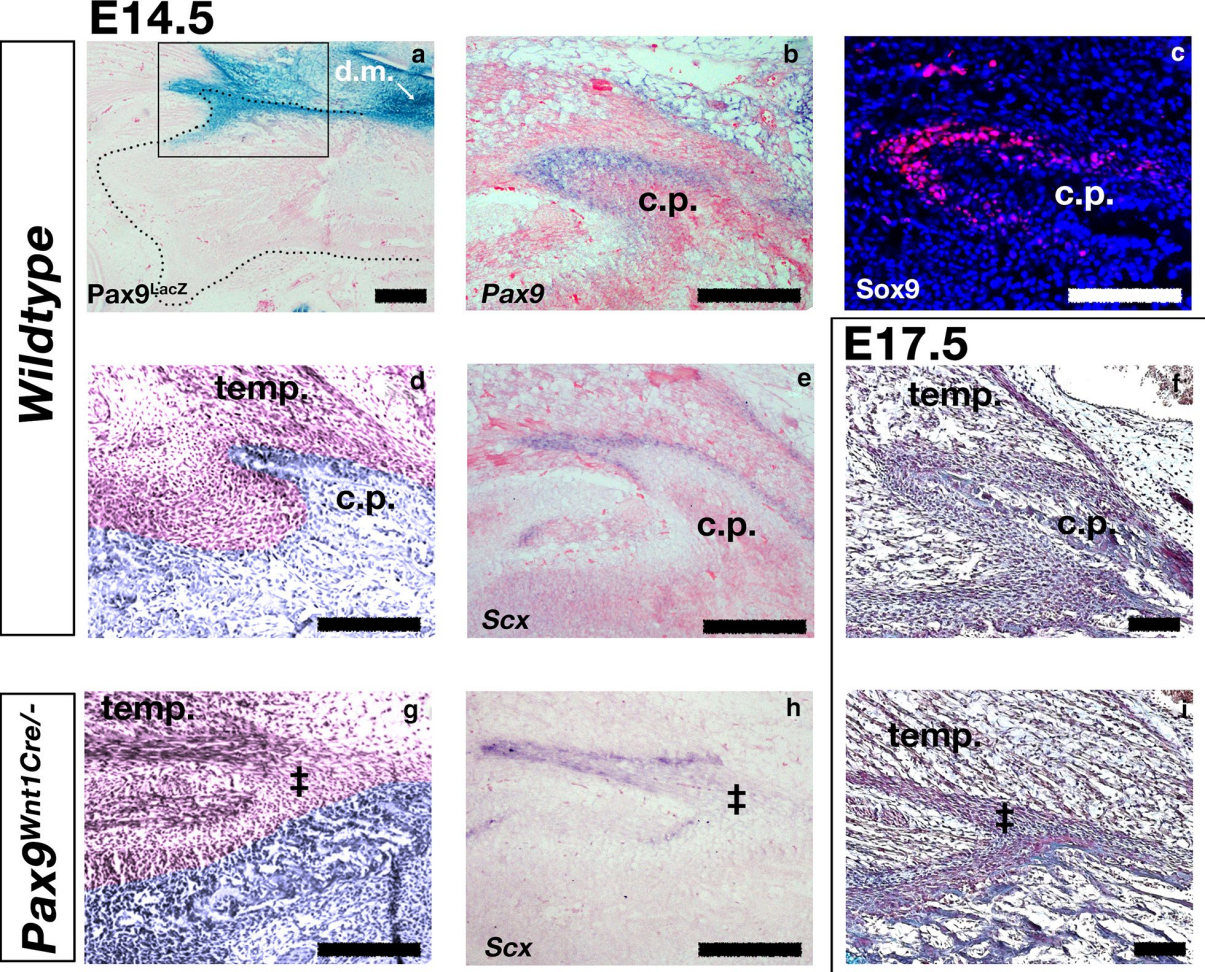
Pax9 in developing mouse dentary coronoid process. **a**, **b** Parasagittal section through proximal mouse mandible at E14.5 to show domain of Pax9 expression. *Pax9* ^*LacZ*^ reporter mouse (**a**) and in situ hybridisation for *Pax9* (**b**) show gene expression in the condensed mesenchyme surrounding the coronoid process. This expression domain is continuous with the Pax9 expressing mesenchyme of the dental field (d.m. in **a**); **c** Sox9 protein, detected by immunofluorescence, is expressed in the same domain around the ossified coronoid process as Pax9; **d**, **g** haematoxylin and eosin staining of the coronoid process at E14.5 after conditional deletion of Pax9 in Wnt1-positive neural crest lineage. The ossified dentary is pseudo-coloured. An ossified coronoid process is observed in wildtype mice (**d**), but not in conditional mutant mice (**g**). **f**, **i** Sirius red/alcian blue trichrome staining shows that the coronoid process fails to develop by E17.5. Loss of *Pax9* expression does not result in loss of the temporalis muscle (**d**, **f**, **g**, **i**). **e**, **h** in situ hybridisation staining for *Scleraxis* (*Scx)* mRNA. The connection between the temporalis muscle and dentary bone is maintained via *Scx* expressing tendon cells which form around an outgrowth of periosteal tissue in place of the coronoid process (*double dagger* in **g**–**i**); *cp* coronoid process; *temp* temporalis muscle; *dm* dental mesenchyme; *Scale bar* in **b**–**i** is 100 µm

It has previously been reported that deletion of *Pax9* in neural crest leads to loss of the coronoid process (Fig. 1d), alongside loss of other structures, including the teeth, alveolar bone, palatal process of the premaxila, and formation of a cleft of the secondary palate [8]. To determine which cells are affected by the loss of *Pax9* gene expression, E14.5 *Pax9*^*LacZ*^ reporter mice were generated. LacZ positive cells were found in the condensed mesenchyme surrounding the coronoid process, which was part of a region of expression continuous with the Pax9+ mesenchyme of the developing tooth (Fig. 2a). This was confirmed by in situ hybridisation (Fig. 2b). Furthermore, due to the long half-life of LacZ, around 30 h in mammalian cells [23], we were able to track the short term fate of these Pax9 expression cells after *Pax9* was no longer evident by in situ hybridisation. Using this method, Pax9^LacZ^ positive cells were observed in the bone of the coronoid process at a time when in situ for *Pax9* mRNA was negative, showing that Pax9 cells give rise to the bone of the process (Fig. 2a). Immunohistochemistry revealed that Sox9 was also expressed in the condensed mesenchyme surrounding the coronoid process (Fig. 2c); this was in the absence of any cartilage growth at this location in the mouse during embryonic development as observed by wholemount alcian blue staining (see Fig. 1) and histological staining (Fig. 2).

To determine if loss of the process in the absence of *Pax9* is due to an intrinsic patterning defect we investigated the dentary of *Wnt1*-*Cre*^*Cre/*+^*;Pax9*^*flox/flox*^ (hence called *Pax9*^*Wnt1Cre/*−^) mutant mice both at the point of ossification of the dentary at E14.5 and once the surrounding muscle tissue had developed at E17.5. At E14.5 the coronoid process was defined and easily identified in Cre negative wildtype (*Pax9*^*Wnt1Cre/*+^) embryos, but was absent from the *Pax9*^*Wnt1Cre/*−^ littermates (Fig. 2d, g). The process was also absent at E17.5 when observed with pico-sirius red, alicain blue trichrome staining (Fig. 2f, i). The temporalis muscle was still present in the null mutant and appeared to form an attachment with fibrous tissue at the superior part of the dentary where the coronoid is normally found. In situ hybridisation for *Scleraxis* (*Scx)* revealed that tendinous attachment between the muscle and bone was maintained in the region (Fig. 2e, h).

### *Pax9* is expressed in the coronoid process of guinea pigs and opossums

**Fig. 3:**
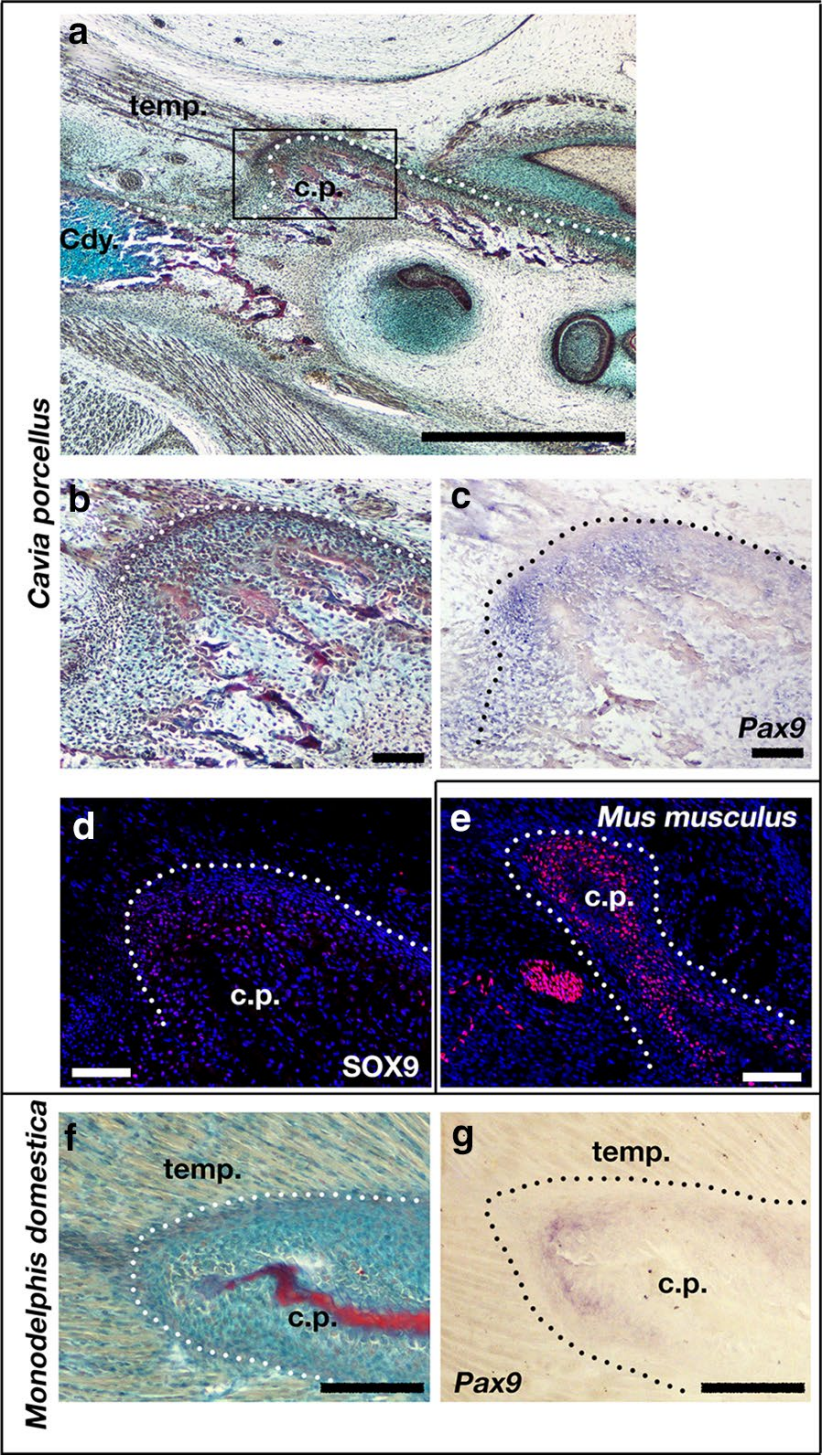
*Pax9* expression in coronoid process of guinea pig and opossum. **a**, **b**, **f** Sirius red/alcian blue trichrome staining of parasagittal section through dentary of E32 guinea pig (**a**, **b**) and P10 opossum (**f**). The rudimentary coronoid process of the guinea pig is associated with a small temporalis muscle containing very few fibres (**a**, **b**) when compared with the large muscle surrounding the process of the opossum (**f**). **c**, **g** In situ hybridisation shows that *Pax9* is expressed around the coronoid process of both the guinea pig (**c**) and the opossum (**g**). **d**, **e**. Immunofluorescence staining shows that Sox9 is weakly expressed around the coronoid process of the guinea pig (**d**) compared to the E15.5 mouse (**e**); *temp* temporalis muscle; cp *coronoid process*; *Cdy* mandibular condyle; *Scale bar* in **a** is 1000 µm; Scale in **b**–**f** is 100 µm

It is demonstrated above that *Pax9* plays a role in establishment of the coronoid process during the ossification of the dentary in the mouse. To investigate whether *Pax9* has a conserved role in initiation of the coronoid process we investigated *Pax9* expression in different mammalian species, guinea pig (*Cavia porcellus*), and early postnatal grey short tailed opossum (*Monodelphis domestica*) (Figs. 1, 3). As a marsupial, opossums are born early compared to placental mammals in their development and the dentary continues to undergo morphogenesis during the first post-natal weeks. Opossum pups do not form a secondary mammalian squamosal-dentary jaw joint, known in humans as the temporomandibular joint (TMJ), until the end of the second postnatal week [24, 25]. This joint is formed in mice by E16.5 [26], thus in this regard opossum pups are equivalent to late stages of embryonic mice. Opossums and guinea pigs were chosen due to the different sizes of their coronoid processes relative to the size of the dentary in the adult, and the fact that they lack of secondary cartilage at the developing coronoid process (Fig. 1), which is similar to the mouse but contrasting to other species such as rats and humas [5].

We have previously suggested that the coronoid process is initially patterned in the guinea pig embryo, but lost or reduced by birth [5]. Trichrome staining demonstrated that at embryonic day 32 (E32), equivalent to mouse E15.5 in terms of craniofacial development [27], the guinea pig coronoid process is present superior to the condylar process and that it serves as the muscle attachment site for the temporalis muscle (Fig. 3). This muscle, however, appears to be relatively small, containing relatively fewer fibres and making only a small surface area connection with the dentary (Fig. 3a), in keeping with the adult muscle, which is smaller as a proportion of total masticatory muscle volume compared with other rodents such as mice, rats and squirrels [28, 29]. In a similar fashion to that seen in the mouse, *Pax9* gene expression was observed around the coronoid process of the developing guinea pig (Fig. 3b, c), while Sox9 protein expression was detected in a similar domain in guinea pig and mouse processes (Fig. 3d, e). Interestingly, the number and intensity of positive cells appeared relatively reduced (Fig. 3d, e), possibly relating to the reduced muscle attachment compared with mice. The size of the process and muscle attachment seen in the guinea pig is in stark contrast to the opossum at postnatal day 10 (P10), where histology demonstrated that many fibres of the large temporalis muscle surrounded the large coronoid process. Despite the differences in the relative size of their coronoid processes both the guinea pig and opossum exhibited similar expression of *Pax9* mRNA in the condensed mesenchyme of the coronoid (compare Fig. 3c, g).

### Reduction in the temporalis muscle results in no change in *Pax9* expression, and a loss of Sox9 expression in the forming coronoid process

**Fig. 4:**
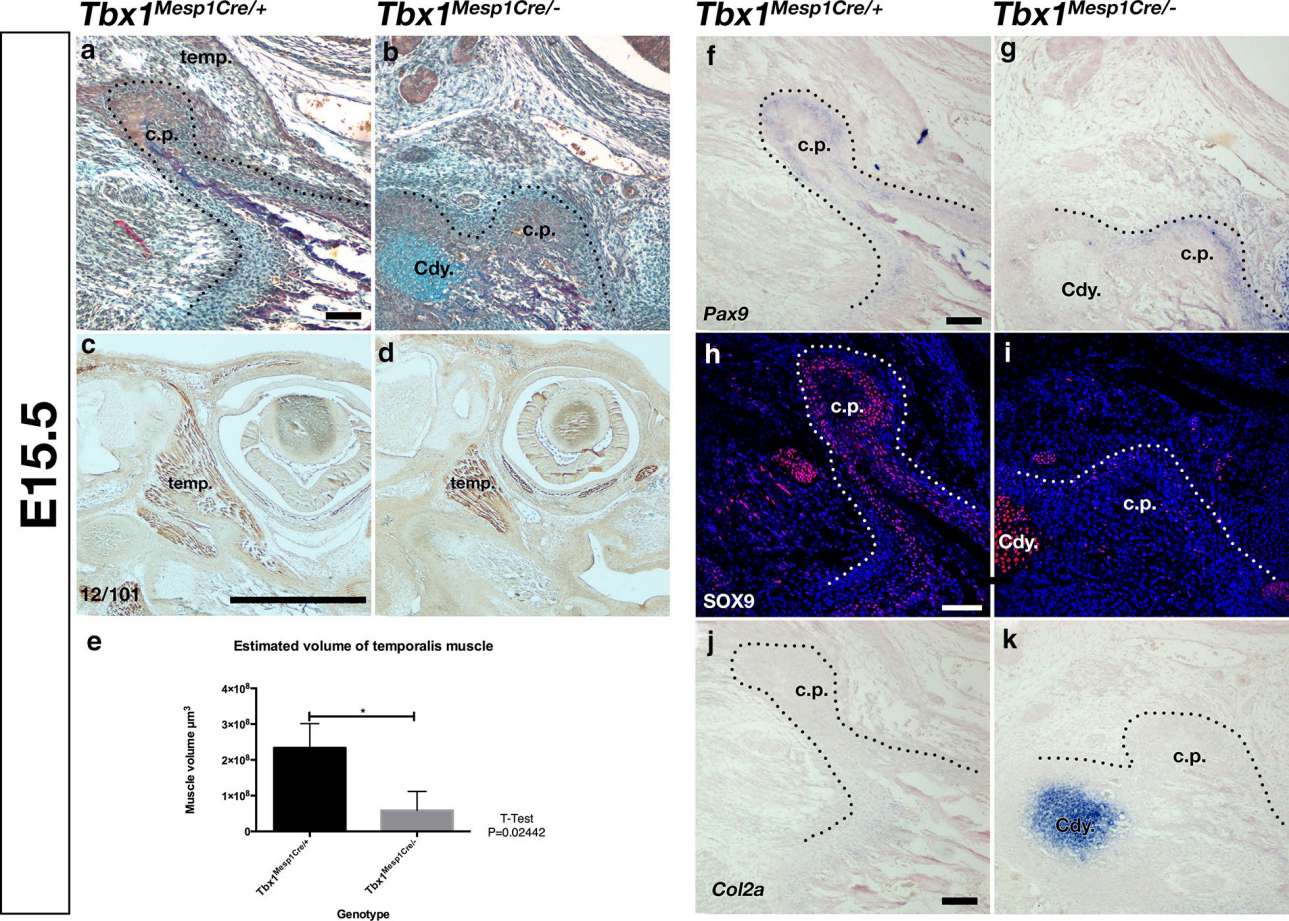
Deletion of *Tbx1* in *Mesp1* lineage mesoderm results in defect in coronoid process growth. **a**, **b** Sirius red/alcian blue trichrome staining of parasagittal section through E15.5 coronoid process of wildtype mouse (**a**) and residual coronoid process of *Tbx1* ^*Mesp1Cre/*−^ conditional mutant mouse (**b**). **c**, **d** Immunohistochemistry at E15.5 for muscle using muscle specific 12/101 antibody demonstrates a reduction in size of temporalis of mutant mice (**d**) compared to wildtype littermates (**c**) **e** Comparison of volume of temporalis muscle in µm^3^, estimated from histological staining (**a**, **b**), demonstrated that the temporalis muscle is significantly reduced in mesoderm specific *Tbx1* mutants compared with wildtype littermates. **f**, **g** In situ hybridisation at E15.5 reveals expression of *Pax9* around the coronoid process is unchanged in *Tbx1* ^*Mesp1Cre/*−^ (**g**) when compared with wildtype littermates (**f**). **h**–**k** At E15.5 Sox9 is detected by immunofluorescence around the E15.5 coronoid process in wildtype mice (**h**), whereas in situ hybridisation for *Col2a* shows that this expression is not associated with cartilage at the coronoid process (**j**). Sox9 expression is reduced in *Tbx1* ^*Mesp1Cre/*−^ mutant mouse coronoid process (**i**), and *Col2a* expression is not present (**k**), while expression of both is maintained in the condylar cartilage; *cp* coronoid process; *Cdy* mandibular condyle; *temp* temporalis muscle; *Scale bar* is 100 µm except **c** and **d** where *bar* is 1000 µm

To confirm that loss or reduction of cranial muscle is not sufficient to prevent the initiation of the mandibular coronoid process, mouse conditional knockouts for *Tbx1* in mesoderm lineage cells (*Mesp1*^*Cre/*+^*;Tbx1*^*flox/flox*^, hence called *Tbx1*^*Mesp1Cre/*−^) were generated. This conditional knockout is known to have a variable level of penetrance, with low levels of *Tbx1* being detected in some mesodermally derived tissues in mutant embryos [19]. To fully determine the level of muscle reduction, samples were collected at E15.5. Histology and immunohistochemistry showed that at E15.5 *Tbx1*^*Mesp1Cre/*−^ mutant mice had a significantly reduced temporalis muscle, but still possessed a coronoid process, though it was of substantially reduced size when compared to wildtype (*Tbx1*^*Mesp1Cre/*+^) littermates (Fig. 4a, b, c, d, e). This rudimentary coronoid was lost later in development [19]. To determine if the expression of *Pax9* and Sox9 in the developing coronoid was dependent upon interaction with the muscle in the mouse, in situ hybridisation for *Pax9* and immunohistochemistry for Sox9 were carried out in *Tbx1*^*Mesp1Cre/*−^ mutants with reduced temporalis muscle size. *Pax9* expression was found in the condensed mesenchyme surrounding the rudimentary coronoid process of the *Tbx1*^*Mesp1Cre/*−^, in a similar pattern and intensity to that observed in *Tbx1*^*Mesp1Cre/*+^ littermates (Fig. 4f, g). However, compared with wildtype littermate, *Tbx1*^*Mesp1Cre/*−^ mutant mice displayed a reduction in Sox9 expression around the vestigial coronoid process, with only a small number of weakly stained cells being present (Fig. 4h, i). As in the wildtype, no *Col2a* expressing cells were observed in the coronoid process (Fig. 4j, k), confirming the lack of secondary cartilage at the coronoid process. In contrast strong expression was observed as expected in the condylar cartilage (Fig. 4k). These data, along side the difference in Sox9 expression between mouse and guinea pig, imply a relationship between Sox9 expression and extrinsic muscle interactions.

**Fig. 5:**
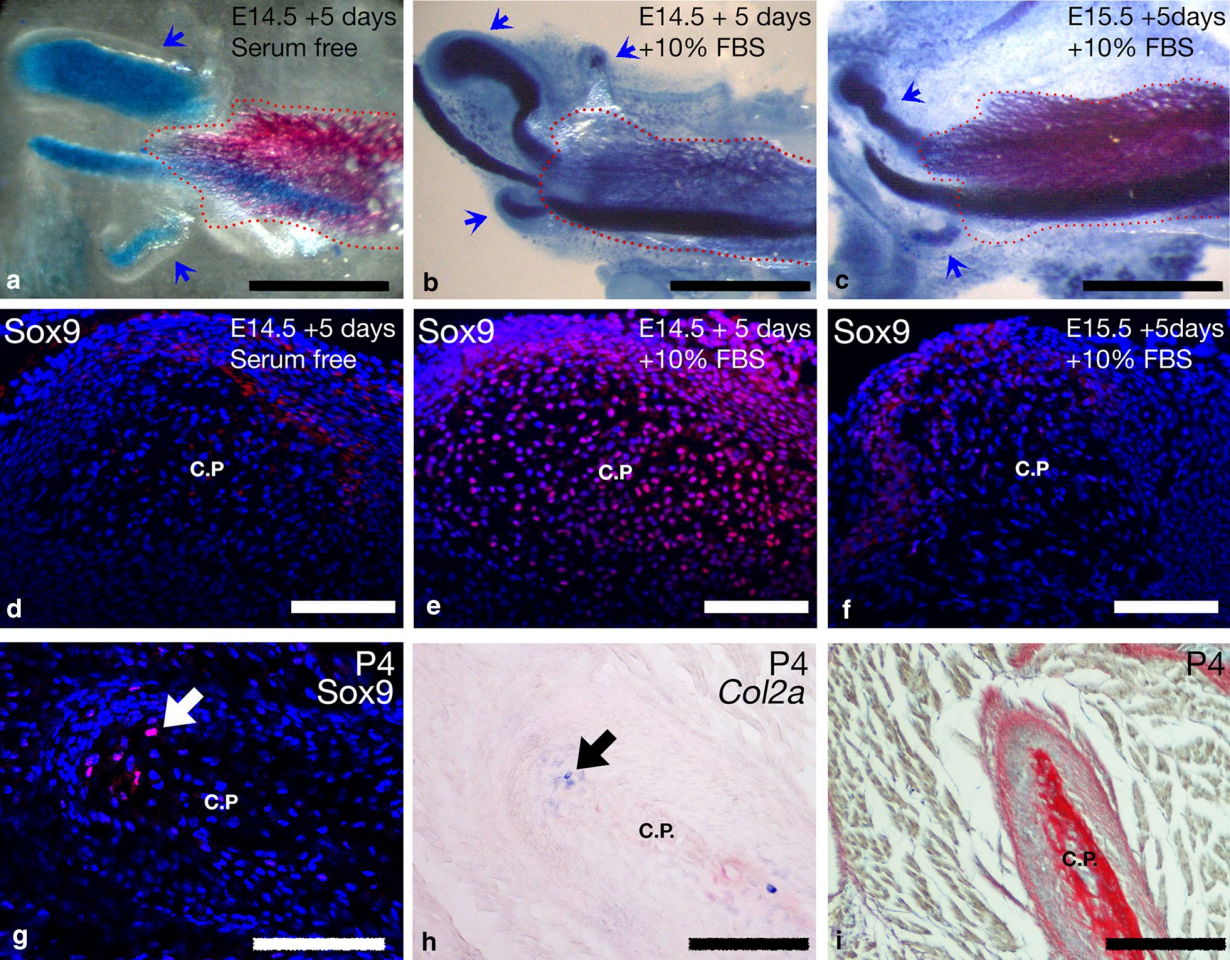
Induction of coronoid secondary cartilage in mice. **a–c** Alizarin red/alcian blue staining of E14.5–E15.5 dentary explant cultures shows that the condylar and angular process cartilages seen in vivo grow after 5 days culture in serum free conditions whereas no coronoid process cartilage is observed (**a**), addition of foetal bovine serum results in the formation of coronoid process secondary cartilage in explants of E14.5 dentary tissue (**b**), but not of E15.5 (**c**). *Blue arrowheads* show location of secondary cartilages. **d–f** Immunofluorescence of Sox9 in histological section of explants demonstrates that Sox9 expression is not expressed within the coronoid position of E14.5 explants without serum (**d**), but is highly expressed in E14.5 explants with serum where cartilage develops (**e**). Sox9 was not expressed in E15.5 explants with serum (**f**). **g–i.** In postnatal day 4 mouse coronoid process immunofluorescence shows Sox9 + cells to be reduced in periosteal cells and irregular within the ossified process (*arrow* in **g**). A few cells in the ossified domain also express *Col2a*, as shown by in situ hybridisation (*arrow* in **h**). No alcian blue stained cartilage is observed in sirius red/alcian blue trichrome staining (**i**); *C.P.* coronoid process; *Scale bar* in **a**–**c** is 1000 µm, and **d**–**i** is 100 µm

Although a secondary cartilage is not found at the coronoid in the mouse, guinea pig and opossum it is found in other species, such as the rat and human. This is particularly interesting given the close phylogenetic relationship of mice and rats. Given the expression of Sox9 in the mouse coronoid process we therefore tested the ability for a secondary cartilage to grow at this location in explant culture in the mouse. We have previously demonstrated an explant culture system for the secondary cartilages of the dentary [6]. In this earlier study, cartilage-like structures were seen superior to the condylar cartilage (see Fig. 4c in Anthwal et al. [6]). We therefore used this culture method to investigate the potential of the coronoid to develop a secondary cartilage when provided with different culture mediums. In the present study mouse E14.5 dentary explants were cultured in BGJb medium with 10 % foetal bovine serum (FBS) or under serum free conditions. Explants were then fixed in 70 % ethanol and wholemount stained for cartilage with alcian blue and mineralised tissue with alizarin red. When cultured in the absence of serum, the condylar and angular secondary cartilages were induced as previously described in 100 % of explant cultures (*n* = 10), but as in vivo, no coronoid cartilage was observed (Table 1; Fig. 5a). Explants cultured with 10 %FBS for 5 days (*n* = 16) showed cartilages at all three processes in at least 75 % of all cultures (Table 1; Fig. 5b). However, when explants were made from E15.5 mouse mandibles (*n* = 42) no coronoid cartilage was induced, while cartilages were induced at the condylar process and angular processes in 93 and 60 % of cases respectively (Table 1; Fig. 5c). The presence and absence of secondary cartilage in these explants is correlated with the expression of Sox9 within the coronoid processes (Fig. 5d, e). These data demonstrate that a secondary cartilage can be induced by extrinsic factors provided in the FBS at the mouse coronoid process in a stage dependant manner, with competency being lost between E14.5 and E15.5. In light of these observations, and the finding that Sox9 is expressed around the coronoid process during development (Fig. 2c), it may be considered surprising that no cartilage is found in the embryonic mouse coronoid. It was therefore hypothesised that the potential for cartilage may only occur once development has progressed further following addition of paracrine and mechanical factors from the mature muscle and surrounding tissue. To investigate this, Sox9 immunohistochemistry and *Col2a* in situ hybridisation was carried out in postnatal mouse coronoid processes. At this stage a few Sox9 and *Col2a* positive cells were observed within the bone matrix; however, no cartilage of the hyaline type typically seen capping the condylar and angular processes was observed by alcian blue staining (Fig. 5g–i).

**Table 1:** Location of secondary cartilages in the mouse dentary following 5 days of explant culture

Location of cartilage	Explant starting age and medium		
	E14.5 BGJb serum free (*n* = 10)	E14.5 BGJb +10 % FBS (*n* = 16)	E15.5 BGJb +10 % FBS (*n* = 42)
Coronoid	0 %	75 %	0 %
Condylar	100 %	100 %	93 %
Angular	100 %	75 %	60 %

## Discussion

**Fig. 6:**
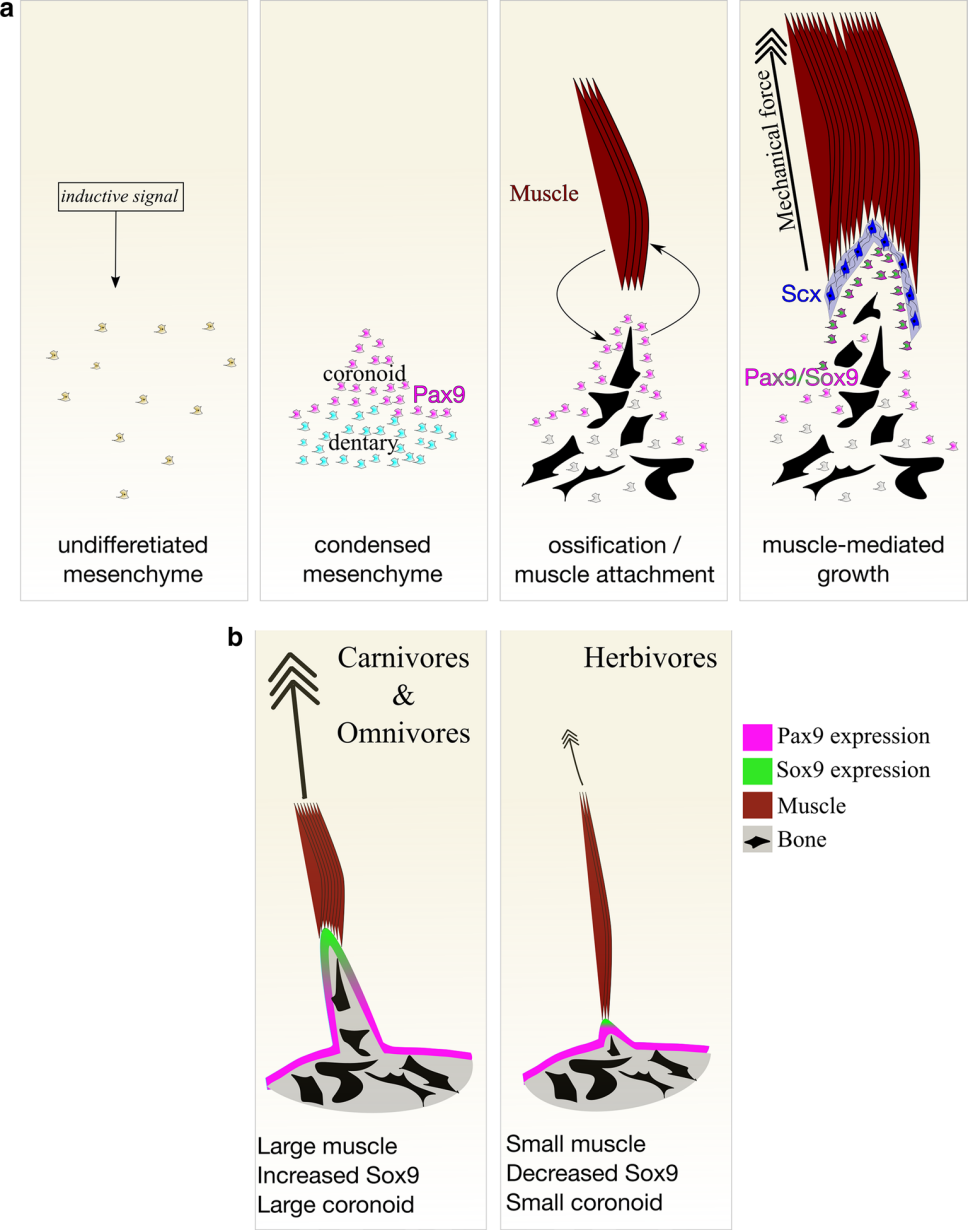
Schematic model of coronoid process development and regulation of growth. **a** An as yet unknown signal induces undifferentiated mesenchyme (*yellow cells*) to condense to form the future dentary bone. The coronoid process is specified by the expression of Pax9 (*magenta*) in the condensed mesenchyme at the superior aspect of the dentary (*cyan*). Interaction between the dentary and the temporalis muscle results in the muscle attachment site forming labelled by *Scx* expression (*blue*) and the dentary begins to ossify. Muscle loading switches on Sox9 expression (*green*) in the Pax9 domain, thereby mediating the expansion of the processes. **b** Proposed model for the role of muscle loading in establishing variation of the coronoid process across species. When compared with omnivore and carnivores, herbivore species have smaller temporalis muscle. This results in a comparatively reduced mechanical load to the coronoid process, a reduced expression of Sox9, and consequently a smaller coronoid process. Schematised plane of section is sagittal

In this study, we demonstrate the role of *Pax9* in the initiation of the coronoid process of the mammalian dentary bone, and demonstrate by use of mutant mice with reduced mesoderm derived jaw muscles that further development of this process from the initial patterning is dependent upon the extrinsic effect of the temporalis muscle (Fig. 6a). We demonstrate that Pax9 is present in the mesenchyme that gives rise to the coronoid process in mammals with different sized coronoid processes: opossum, mouse and guinea pig. We also demonstrate that Sox9, a transcription factor normally associated with chondrogenesis in the skeleton, is expressed in the developing coronoid process in species where no cartilage is found, and that this expression is dependent on interaction with the temporalis muscle (Fig. 6b).

### A developmental programme involving *Pax9* is required for coronoid initiation independent of mechanical force

The coronoid process of the dentary bone is an important feature of the mammalian mandibular skeleton. The emergence of the coronoid process in the fossil record of mammal-like reptiles precedes that of the definitive mammalian jaw joint [2] and is associated with a transfer of the insertion of the external mandibular adductor muscle (the precursor of the temporalis muscle) from the chondrocranium to the intramembranous dentary [30]. It allows for increased bite force and has played an important role in the evolution of the external adductor jaw musculature and hence in the evolution of mammals [30, 31]. The study of development in transgenic mice gives insight into these changes since it allows for the identification of the genetic, developmental, and morphogenic basis upon which form is based. A gene important to this change is *Pax9*.

*Pax9* is known to be a key gene during odontogenesis, where it is expressed in the condensing mesenchyme of the future tooth [32–35]. We demonstrate that the expression domain of *Pax9* in the coronoid process is continuous with the dental mesenchyme. *Pax9* also plays a key role in the development of the secondary palate [34, 36], another intramembranous bone that is a mammalian feeding specialisation. The role of *Pax9* in tooth development is evolutionary ancient, demonstrated by a conserved role in odontogenesis in fish [37], reptiles [38], as well as mammals. In the dental mesenchyme *Pax9* has been shown in mouse explant culture experiments to be expressed in response to the mechanical compression of tissue due to opposing attraction and repulsion signals [39]. Since the *Pax9* domain in mammals is found at a region of muscle interaction, the question of whether a similar mechanism is at play in the coronoid process has previously not been addressed. Given the location of the tissue at the boundary between osteogenic and myogenic tissues, it is possible that as yet unidentified signals act upon competent mesenchyme.

The coronoid process and secondary palate are evolutionary novelties, and as such may have arisen by the co-option of genetic pathways into novel developmental processes [40]. The *Pax9* expressing dental domain is continuous with that of the coronoid and palate and these tissues, teeth, coronoid, palate, all share a common neural crest origin [41–44]. It can therefore be postulated that *Pax9* from the dental mesenchyme has been co-opted into the coronoid and palatal programmes. It would be interesting to investigate whether *Pax9* has an as yet unknown role in membranous bone development in the head of non-mammalian vertebrates. It would also be of interest to investigate the molecular interactions of *Pax9* to fully describe its role in membranous ossification.

### The extrinsic effects of the muscle of jaw closure are required for growth but not initiation of the coronoid, and Sox9 is a potential mediator of this mechanism

The role of extrinsic muscle interaction, such as mechanical force, in the initiation and development of the mandibular coronoid process has been a question of debate for some time. Early surgical experiments, such as those by Washburn in rats, and Soni and Malloy in guinea pigs, indicate that the mechanical force of the temporalis is required for the growth of the coronoid process [16, 17]. This is supported by studies, including the present study, in mouse mutants with reduced or absent temporalis muscles [18, 19, 45]. Rot-Nikcevic and colleagues highlight the hypothesis that the coronoid itself has its origins within the myogenic lineage [18]. Our data do not support this conclusion, as deletion of Pax9 in neural crest lineage cells, which form the hard and connective tissues of the mandible but not the musculature, leads to a complete failure in coronoid induction. In contrast, reduction of muscle by deletion of Tbx1 in mesoderm results in the formation of a rudimentary process. Furthermore, relative *Pax9* expression does not vary between opossum, mouse, and guinea pig, three species with variable bite force and adult coronoid process length. Therefore, our data support the view that muscle attachment is required for the growth of the process and offers Sox9 as a potential mediator for this growth. Expression of Sox9 is reduced when muscle attachment is reduced, as in the case when comparing the wildtype mouse coronoid process with that of either the guinea pig or *Tbx1*^*Mesp1Cre/*−^ mutant mouse. The precise nature of the extrinsic effect of the muscle attachment is still unclear. It seems likely that mechanical stimulation plays a role; however, paracrine signalling from the muscle to the ossifying bone may be involved. Indeed, adult skeletal muscle is known to secrete osteogenic factors Igf1 and Fgf2 [46, 47], and the connection between the deltoid muscle and the humerus via the Scx+ tendon in the limb is a consequence of signalling cross talk between all the three tissues [48]. To determine whether paracrine or mechanical stimulation is the predominate signal for coronoid growth, and possibly for Sox9 expression, further studies will be required. These could include in utero paralysis of embryos, although this is technically challenging, and targeted deletion of candidate paracrine factors such as Igf1 or Fgf2 from the muscle.

### The presence of secondary cartilage may be reliant upon mechanical stimulation in concert with paracrine signals

Given the role Sox9 has in cartilage development [49, 50] its expression at the coronoid process of mice and guinea pigs is surprising as we are unable to detect cartilage at this location during embryonic development in vivo. It may be hypothesised that Sox9 expression is broadly expressed at its onset, becoming restricted and elevated sufficiently to form secondary cartilage if the required stimulation from the muscle is present. However, if this were to be the case, then dentary secondary cartilage would not form at all in mouse models lacking muscle. The muscle-free *Myf5/MyoD* double knockout mice do develop condylar cartilage [18], as do the *Tbx1*^*Mesp1Cre/*−^ mice presented here (Fig. 4b), indicating that at least for the condylar cartilage muscle is not necessary for secondary chondrogenesis. Other authors have referred to secondary cartilages in the mouse coronoid process [18, 51, 52]; however we and others are unable to observe such cartilages during embryonic development, neither by histological staining nor by gene expression [53]. Beresford suggests that this is due to the coronoid cartilage being very transient and therefore easily missed, although no indication is given for when during development this transient cartilage is observed [52]. We were able to observe the occasional Sox9 +/*Col2a* + cell within the bone matrix, but crucially not in the ossifying mesenchyme, of postnatal coronoid processes. These may be the proposed secondary cartilages of Beresford, though it is to be concluded that these cartilage cells are quite different to the cartilages of the condylar and angular process, which form during embryonic development and undergo endochondral ossification thus forming the processes. The presence of Sox9 during development and the ability of cultured dentaries to develop stable coronoid secondary cartilages in the presence of serum might support the idea that the coronoid can form cartilages in a similar manner to the condylar and angular processes. Secondary cartilages have been observed in the coronoids of *PTHrP* knockout mice [54]. Furthermore PTHrP is not expressed in the quadratojugal of the chick embryo, which develops a secondary cartilage [55]. Together these data demonstrate that this pathway may be involved in regulating the presence or absence of cartilage across species.

It is possible that the extent of muscle loading influences whether a species has a secondary cartilage at the coronoid or not. In mice it could be supposed that the absence of the coronoid cartilage is due to reduced mechanical force acting upon the coronoid in contrast to the rat, which in adults has a slightly larger coronoid and a larger muscle. However, this does not take into account the differences in scale between the mouse and rat. The reduced expression of Sox9 in the coronoid process in response to a reduction in muscle, observed in the present study in the guinea pig and in mutant mice, seems to support the hypothesis that cartilage and mechanical loading are related. However, no cartilage has been observed in the opossum [24], where a large and powerful temporalis muscle is associated with a relatively large coronoid process, suggesting that, at least in marsupials, such forces are not able to cause cartilage induction. Therefore, it could be that mechanical stimulation alone is insufficient to form a coronoid secondary cartilage. That we are able to induce cartilage in explants probably relies on the up-regulation of Sox9 observed at the coronoid. Furthermore, the fact that these cartilages were dependant on the addition of serum suggests that induction is at least in part a consequence of paracrine signalling in species where secondary cartilages are observed. That our mouse explants fail to induce a coronoid process after E15.5 suggests that the receptor for any paracrine factors is down-regulated later in mouse development, possibly explaining why no secondary cartilage develops. It can be hypothesised that no such down regulation occurs in those species with a coronoid secondary cartilage, or that the dynamics of the paracrine signalling differs such that visible cartilage is established. The identity of this signal can only be speculated; however, candidates would include members of the Fgf and Bmp signalling families, thanks to their roles in secondary cartilage induction in the chick [56], and possibly members of the PTHrP pathway, for reasons explained above. These interactions are still unclear.

The role of mechanical force in the induction of secondary cartilages has been studied in birds to a greater depth than in mammals. In the chicken (a galiforme), secondary cartilages develop from *Runx2* positive cells of the periosteum of the quadratojugal bone in response to mechanical stimulation of the quadratojugal-quadrate joint [55, 57]. A secondary cartilage is also seen at the insertion site of the jaw adductor muscle, the analogous but not homologous site to the mammalian coronoid process. Species such as ducks that take loading strain on their lower beaks during feeding develop a secondary cartilage at this location, whereas galiformes like the chicken and quail that peck food do not [56]. In these birds Sox9 and other chondrogenic factors are missing from those species without a secondary cartilage, and quail-duck NCC xenografts (quucks) demonstrate that the extrinsic effect of muscle alone is not sufficient to induce cartilage in non-competent tissue [56]. Interestingly, the muscle in these quucks is reduced in size compared to control ducks, possibly demonstrating a feedback from the quail derived bone to the duck muscle [58]. It is interesting that the situation in birds, where by the presence of the secondary cartilage is correlated with the expression of Sox9, contrasts to the mammalian situation where the expression of Sox9 in the coronoid process is observed in the absence of secondary cartilage and the initiation of secondary cartilage at the other processes is independent of muscle loading [6, 18, 59]. These differences support the notion that secondary cartilages in birds and mammals are not homologous but homoplastic, agreeing with the fact that secondary cartilages are observed in two species of teleost, dinosaurs, birds, and mammal, but not in reptiles [60].

### Species differences in coronoid process size are influenced by extrinsic muscle

One of the aims of this study was to try and understand the mechanisms by which variation in the size and shape of the mammalian coronoid process is established. As early as 1947 Washburn suggested that the variation in coronoid form was due to intrinsic patterning of this element, rather than being the consequence of biomechanical interaction [16]. We have previously demonstrated that the proximal mandibular processes are patterned prior to the onset of ossification and muscle action [6]. It has previously been suggested that Pax9, along side other factors including Prx1/2, Gsc, Dlx5, and Bmp2/4 may play a role in the intrinsic growth of the coronoid [5, 9, 61–64]. We demonstrate here that Pax9 expression is not lost in the absence of muscle action acting up on the coronoid process, and that Pax9 is expressed in the developing coronoid of animals with varying coronoid sizes and correlating muscle size. Therefore, we suggest that Pax9 is not the factor controlling the intrinsic difference in coronoid size observed between species, rather it is a factor controlling the intrinsic ability of the dentary bone to initiate the coronoid process. Sox9 may be acting to then control size, since its expression is reduced in the guinea pig coronoid process compared to that of the mouse. Expression levels of Sox9 in mice are reduced in the absence of muscle, indicating that Sox9 expression is dependent on extrinsic stimulation. Importantly, conditional deletion of *Sox9* in neural crest cells results in a loss of the coronoid process at E15.5 [7, 65], highlighting the essential role of Sox9 in coronoid development. Taken together these data suggest that the size of the coronoid may be dependant upon the regulation of intrinsic Sox9 levels within the coronoid process by varying muscle attachment size via biomechanical and/or paracrine signalling. The current study does not take into account the nature of the mechanical load of the muscle in terms of the direction and frequency of muscle contraction. It may be that these factors account for aspects of coronoid growth as suggested by other authors, and that the proposed molecular regulators of coronoid size are modulated by these variables in addition to the size of muscle load. The precise natures of the variations determining coronoid size and shape are still unknown, however, the direction of and size of the mechanical load acting alongside signalling between the muscle and bone may have a greater effect upon the shape and size of the bone than intrinsic programming.

## Conclusions

The wide range of dentary forms observed in the mammalian clade reflects the diversity of dietary niches occupied by mammals. By combining the tools available in mouse genetics with comparative developmental biology, the current study proposes that the induction of a coronoid process of the mandible is intrinsic to the dentary bone, with *Pax9* playing a key role. The diversity in size of the mandibular coronoid across species is not due to intrinsic factors acting to alter patterning, instead the extrinsic effect of muscle directly influences the size of the process as it forms though regulation of membranous ossification, likely via Sox9. This represents an as yet described role for Sox9 in skeletal development independent of cartilage.

Our findings highlight the advantage of using a combination of mouse knockouts and comparative developmental biology to gain an understanding of the mechanisms of mammalian evolution.

## Abbreviations

BGJb: BGJ medium, Fitton-Jackson modified
Bmp2: bone Morphogenetic Protein 2
Bmp4: bone Morphogenetic Protein 4
cp: coronoid process
Cdy: mandibular condyle (condylar process)
Col2a: type 2 collagen, alpha 1
DAPI: 4′,6-diamidino-2-phenylindole
DEPC: diethylpyrocarbonate
Dlx5: distal-less homeobox 5
E: embryonic day
EDTA: ethylenediaminetetraacetic acid
FBS: foetal bovine serum
Gsc: goosecoid homeobox protein
Mesp1: mesoderm posterior 1 homolog
mRNA: messenger RNA
Myf5: myogenic Factor 4
MyoD: myogenic Differentiation
NCC: neural crest cells
P: postnatal day
Pax9: paired box gene 9
PBS: phosphate buffered saline
PCR: polymerase chain reaction
PFA: paraformaldehyde
Prx1/2: paired related homeobox 1/2
PTHrP: parathyroid hormone-related protein
RNA: ribonucleic acid
Runx2: runt-related transcription factor 2
Scx: scleraxis
Sox9: SRY-box gene 9
Tbx1: T-box protein 1
Temp: temporalis muscle
Wnt1: wingless-type MMTV integration site family, member 1
X-Gal: 5-bromo-4-chloro-3-indolyl-β-D-galactopyranoside
